# Comparative *in silico* analysis of SSRs in coding regions of high confidence predicted genes in Norway spruce (*Picea abies*) and Loblolly pine (*Pinus taeda*)

**DOI:** 10.1186/s12863-015-0304-y

**Published:** 2015-12-26

**Authors:** Sonali Sachin Ranade, Yao-Cheng Lin, Yves Van de Peer, María Rosario García-Gil

**Affiliations:** Department of Forest Genetics and Plant Physiology, Umeå Plant Science Centre, Swedish University of Agricultural Sciences, SE-901 83, Umeå, Sweden; Department of Plant Systems Biology (VIB) and Department of Plant Biotechnology and Bioinformatics, Ghent University, Technologiepark 927, 9052 Ghent, Belgium; Genomics Research Institute, University of Pretoria, Hatfield Campus, Pretoria, 0028 South Africa; Bioinformatics Institute Ghent, Ghent University, 9052 Ghent, Belgium

**Keywords:** Norway spruce, *Picea abies*, Loblolly pine, *Pinus taeda*, Simple sequence repeats (SSR), Microsatellites, High confidence genes

## Abstract

**Background:**

Microsatellites or simple sequence repeats (SSRs) are DNA sequences consisting of 1–6 bp tandem repeat motifs present in the genome. SSRs are considered to be one of the most powerful tools in genetic studies. We carried out a comparative study of perfect SSR loci belonging to class I (≥20) and class II (≥12 and <20 bp) types located in coding regions of high confidence genes in *Picea abies* and *Pinus taeda*. SSRLocator was used to retrieve SSRs from the full length CDS of predicted genes in both species.

**Results:**

Trimers were the most abundant motifs in class I followed by hexamers in *Picea abies*, while trimers and hexamers were equally abundant in *Pinus taeda* class I SSRs. Hexamers were most frequent within class II SSRs followed by trimers, in both species. Although the frequency of genes containing SSRs was slightly higher in *Pinus taeda*, SSR counts per Mbp for class I was similar in both species (*P*-value = 0.22); while for class II SSRs, it was significantly higher in *Picea abies* (*P*-value = 0.00009). AT-rich motifs were higher in abundance than the GC-rich motifs, within class II SSRs in both the species (*P*-values = 10^−9^ and 0). With reference to class I SSRs, AT-rich and GC-rich motifs were detected with equal frequency in *Pinus taeda* (*P*-value = 0.24); while in *Picea abies*, GC-rich motifs were detected with higher frequency than the AT-rich motifs (*P*-value = 0.0005).

**Conclusions:**

Our study gives a comparative overview of the genome SSRs composition based on high confidence genes in the two recently sequenced and economically important conifers and, also provides information on functional molecular markers that can be applied in genetic studies in *Pinus* and *Picea* species.

**Electronic supplementary material:**

The online version of this article (doi:10.1186/s12863-015-0304-y) contains supplementary material, which is available to authorized users.

## Background

Microsatellites or simple sequence repeats (SSRs) are DNA sequences consisting of 1–6 bp tandem repeat motifs widely distributed in the coding and non-coding parts of the genome [1], resulting from DNA-polymerase slippage during replication and unequal recombination [2]. Microsatellites are co-dominant, multi-allelic and reproducible besides having high mutation rates [3]. Microsatellite analysis is fast and cost effective with the present technology [4–6]. Due to these properties, they are considered to be one of the most powerful tools for analysis of genetic biodiversity [7], and are also widely used as molecular markers in marker-assisted selection [8], mapping and phylogeny [9].

SSRs are classified according to their length into class I composed of those with ≥20 bp repeats and class II containing repeats from 12 to 20 bp. Class I motifs are of prime importance from the point of view of applicability of the SSRs as markers due to their higher polymorphic nature compared to class II SSRs [10]. SSRs are also grouped into three types based on their complexity - perfect, imperfect and compound SSRs. Perfect SSRs are continuous repetitions of motifs without any interruption by any base (e.g. (AT)_20_), while in an imperfect SSR the repeated sequence is interrupted by different nucleotides that are not repeated (e.g., (AT)_12_GC(AT)_8_). Compound SSRs contain two adjacent distinct SSRs (e.g. (AT)_7_(GC)_6_).

Norway spruce (*Picea abies*) and Loblolly pine (*Pinus taeda*) are two important conifer species from an economical and ecological point of view. With the availability of the *Picea abies* [11] and Loblolly pine [12] genome assemblies, comparison between their genomes on various aspects is feasible and they have become the conifer model species to conduct further comparative research in gymnosperms [13, 14]. The distribution of long terminal repeat-retrotransposons (LTR-RTs: Ty1/Copia and Ty3/Gypsy) was similar in *Picea* (*Picea abies*) and *Pinus* (*Pinus sylvestris*) [11]. In this context the current analysis updates on the comparative distribution of the SSR loci within the two species.

There are few investigations, which have reported the analysis of EST-SSRs (Expressed sequence tags) in *Picea* spp. [15, 16] and *Pinus taeda* [15, 17–19]. Dimers were detected as the most abundant repeat motifs followed by trimers and hexamers in a majority of these analyses, similar to our earlier comparative study among gymnosperm tree species, which was somewhat limited by the data availability and the study was conducted only at the genus level [14]. Fluch et al. [16] is the only EST-SSR study so far conducted on *Picea abies* and this investigation reports the presence of trimers > pentamers > hexamers in the order of frequency of occurrence. In the current work, we carried out a comparative study of perfect SSRs belonging to the class I and class II types in *Picea abies* and *Pinus taeda* based on coding regions of genes predicted with high confidence CDS) [20]. As compared to previous studies in *Picea* and *Pinus*, our approach allows counting the precise numbers of all repeats motifs across the coding part of the genome, and it is expected that some degree of inconsistency would exist on the estimation of the number of class I SSRs with reference to those reported in previous studies on the basis of the data source and the methodology. We have considered only the high confidence full length genes (CDS) for detection of the repeat motifs and thus the detected loci could serve as robust molecular markers. Genic SSRs have advantages over the genomic SSRs as the putative function of the particular gene is known and they are highly transferrable across species [21]. The aims of this study are: (i) to analyse SSR motifs to identify the species-specific characteristics to gain insights into *Pinaceae* genome composition and (ii) to deliver a list of primers for the development of SSR molecular markers located in expressed genes, which can be applied to species of both genera, *Pinus* and *Picea*, for a range of different genetic studies such as population genetic studies, paternity analysis, genotyping, genetic mapping, molecular evolution and hybrid selection [22].

## Methods

### Genomic resources and procedure

Full length CDS of genes predicted with high confidence from *Picea abies* (26,437 genes) [11], (http://congenie.org/) and *Pinus taeda* (34,059 genes) [20] were included for the detection of SSRs in this work. SSRLocator [23] was used to retrieve the perfect SSR markers belonging to class I (≥20 bp) and class II (≥12 and <20 bp) in both species. SSRLocator was used with the following settings for class I SSRs, SSR repeat motifs and number of repeats as the calculated parameters, monomer-20, dimer-10, trimer-7, tetramer-5, pentamer-4, hexamer-4, heptamer-3, octamer-3, nonamer-3 and decamer-2 [10]. Likewise, following settings were used to detect class II SSRs - monomer-12, dimer-6, trimer-4, tetramer-3, pentamer-3, hexamer-2, heptamer-2, octamer-2 and nonamer-2. Since the class II search also retrieved the class I SSRs, the data was filtered for the redundant results with help of SQL queries. While recording the count of a particular repeat motif, circular permutations and/or reverse complements of each other were clustered together (e.g. AC = GT = CA = TG, ACG = CGA = GCA = TGC = GCT = CGT = AGC = TCG = CAG = GTC = CTG = GAC and AAC = ACA = CAA = TTG = TGT = GTT) [15]. Along with the *in silico* detection of the SSRs, SSRLocator provides list of putative primer pairs which are represented in the Additional file 1. Mononucleotides were included only for the calculation of counts per Mbp (Table 1) but were excluded from rest of the analysis to facilitate the comparison of the results with most other studies which did not consider the analysis of mononucleotides [14, 15, 19, 24, 25], as mononucleotide repeats can be difficult to accurately assay [26]. Moreover mononucleotides were excluded from this study also because of the possibility of sequencing or assembly errors [27, 28]. Blast2GO analysis [29] was performed for class I (≥20 bp) described as more efficient molecular markers [10].

**Table 1:** Counts per Mbp for class I and class II SSRs in *Picea abies* and *Pinus taeda*

	*Picea abies*		*Pinus taeda*	
No. of genes considered for the analysis	26,437		34,059	
	Class I SSRs	Class II SSRs	Class I SSRs	Class II SSRs
No. genes with SSRs	240	11380	337	14967
Motif length^a^ (bp)	23.7 (4.6)	12.7 (1.6)	22.7 (4.1)	12.7 (1.6)
SSR counts per Mbp	54.7	1,768.2	42.7	1,541.9
No. genes with class I and class II SSRs	149		203	

^a^Standard deviation for SSR length is shown in between parenthesis

### Statistical analysis

We carried out a contingence *χ*2 test for heterogeneity of microsatellite counts (motif counts/total EST-fraction in Mbp) among different counts per Mbp within and between species. A *t*-test was applied to compare means among two groups of data. Statistical analyses were all carried out using the R software package [30].

## Results

### Number of genes containing SSRs and motif size

The percentage of genes containing class I and class II SSR loci in *Picea abies* was found to be 0.9 and 43 %, respectively; while in *Pinus taeda* it was 1 and 44 %, respectively. The percentage of genes containing both class I and class II loci was found to be 0.6 % in both species. Although the frequency of genes containing SSRs was similar in both species, counts per Mbp for class II SSRs was higher in *Picea abies* (chi-square = 15.4, *P*-value = 0.00009), while for class I the difference was not significant (chi-square = 1.5, *P*-value = 0.22) (Table 1). Motif lengths were significantly larger in *Picea abies* for class I motifs (*P*-value = 0.006), while lengths were identical in both species for class II SSRs.

### SSR frequency

Trimers were the most abundant motifs in class I SSRs in *Picea abies* (chi-square = 12.9, *P*-value = 0.0003), while trimers and hexamers were equally abundant in *Pinus taeda* (chi-square = 0.04, *P*-value = 0.95). Hexamers were significantly more abundant SSR motifs in class II SSR in both species (*Picea abies*, chi-square = 308, *P*-value = 0; *Pinus taeda*, chi-square = 446, *P*-value = 0) (Table 2). In *Picea abies*, the order of abundance in class I SSRs was trimers > hexamers > decamers, while in class II it was hexamers > trimers > heptamers. Likewise, in *Pinus taeda* the order was trimers = hexamers > dimers/decamers in class I SSRs, while it was hexamers > trimers > heptamers in the class II SSR motifs.

**Table 2:** Counts per Mbp of different SSR motifs for class I and class II SSRs in *Picea abies* and *Pinus taeda*

	*Picea abies*		*Pinus taeda*	
Motif	Counts per Mbp for class I SSRs	Counts per Mbp for class II SSRs	Counts per Mbp for class I SSRs	Counts per Mbp for class II SSRs
Monomer	0.0	8.6	6.2	39.7
Dimer	0.0	11.9	7.9	29
Trimer	36.4	409.6	11.4	231.3
Tetramer	0.0	43.5	0.9	70
Pentamer	0.7	6.4	2.1	13.6
Hexamer	11.5	1,088.0	11.1	959.8
Heptamer	0.5	120.0	0.8	143.9
Octamer	0.0	39.5	0.4	48.2
Nonamer	1.1	49.2	0.4	46.1
Decamer	4.5	0.0	7.7	0

With reference to class I trimers, AGG/CCT and ACG/CGT were both equally abundant and together were the most abundant motifs in *Picea abies* (chi-square = 4, *P*-value = 0.05). Likewise, in *Pinus taeda*, AAT/ATT, AAG/CTT, AGG/CCT and ACG/CGT motifs were equally abundant and together were the most abundant class I trimer motifs (chi-square = 6.3, *P*-value = 0.01) (Table 3). Similarly, regarding class II motifs, AAG/CTT, AGG/CCT and ACG/CGT motifs were significantly the most frequent in *Picea abies* (chi-square = 64.5, *P*-value = 0), and AAG/CTT, AGG/CCT, ACG/CGT and ACT/AGT motifs were the most abundant in *Pinus taeda* (chi-square = 54, *P*-value = 0) (Table 3). While comparing both species, the ranking of the most abundant motifs is not the same for the class I motifs, but is very similar for class II motifs. The total count per Mbp was significantly higher in *Picea abies* in both classes (class I, chi-square = 13.1, *P*-value = 0.0003; class II, chi-square = 49.7, *P*-value = 0).

**Table 3:** Counts per Mbp of trimer motifs for class I and class II SSRs in *Picea abies* and *Pinus taeda*

	*Picea abies*		*Pinus taeda*	
Motif	Counts per Mbp for class I SSRs	Counts per Mbp for class II SSRs	Counts per Mbp for class I SSRs	Counts per Mbp for class II SSRs
ACG/CGT	9.1	87.5	2	46.3
ACT/AGT	1.6	57.7	0.2	35
AAC/GTT	0.3	17.8	0	20
AAG/CTT	5.4	110.9	2.4	50
AAT/ATT	0.3	12.1	2.9	15.9
ACC/GGT	2.1	21.9	1	16.9
AGG/CCT	14.9	87.7	2.3	40.3
CCG/CCG	2.7	14.1	0.6	6.9

In both species the hexamer abundance in class I SSR was similar (Table 4). However, the most abundant motif type differenced among both species, in *Picea abies*, AAACCG was the most abundant, while AACGGT was the most frequent in *Pinus taeda*. With reference to class II hexamers, AACGGT was the most abundant motif type in both species followed by AACCGT in *Picea abies*, which was fourth in *Pinus taeda*; likewise, the fourth most abundant motif in *Picea abies* (AAACGT) was the second most frequent motif in *Pinus taeda* (Table 4). Furthermore, within the class I and II hexamers, total counts per Mbp were higher in *Picea abies*, although the differences were not statistically significant between the two species.

**Table 4:** Counts per Mbp of first two abundant hexamers motifs for class I and class II SSRs in *Picea abies* and *Pinus taeda*

*Picea abies*				*Pinus taeda*			
Motif	Counts per Mbp for class I SSRs	Motif	Counts per Mbp for class II SSRs	Motif	Counts per Mbp for class I SSRs	Motif	Counts per Mbp for class II SSRs
AAACCG	1	AACGGT	88.4	AACGGG	1.1	AACGGT	69.9
AACCCG	0.9	AACCGT	68.8	AAGGGT	1	AAACGT	59.5
AACCGG	0.9	AAAGGT	64.8			AAAGGT	56.7
ACCCCG	0.9	AAACGT	62.5			AACCGT	53.3

### AT-rich and GC-rich motifs

The differential counts of nucleotides per Mbp for class I and class II SSRs revealed that AT-rich motifs were more abundant within the class II SSRs in both species (*Picea abies*, chi-square = 28.6, *P*-value = 10^−9^; *Pinus taeda*, chi-square = 173, *P*-value = 0) (Table 5). Moreover, AT- and GC-rich motifs were equally abundant in class I SSRs in *Pinus taeda* (chi-square = 1.4, *P*-value = 0.24), while GC rich motifs showed higher frequency per Mbp in the class I SSRs in *Picea abies* (chi-square = 12.2, *P*-value = 0.0005). Differential G + C nucleotide count per Mbp was higher than that of A + T in the class I SSRs in *Picea abies* (chi-square = 4.3, *P*-value = 0.04), but the difference between both categories was not significant in *Pinus taeda* (chi-square = 3.3, *P*-value = 0.07). The differential A + T count per Mbp was higher in class II SSRs in both species (*Picea abies*, chi-square = 56.5, *P*-value = 0; *Pinus taeda*, chi-square = 239, *P*-value = 0).

**Table 5:** Differential counts per Mbp of nucleotides in repeat motifs for class I and class II SSRs in *Picea abies* and *Pinus taeda*

	*Picea abies*		*Pinus taeda*	
Nucleotides	Counts per Mbp for class I SSRs	Counts per Mbp for class II SSRs	Counts per Mbp for class I SSRs	Counts per Mbp for class II SSRs
AT-rich	12.8	791.2	20.8	818.6
GC-rich	37.5	542.6	14.3	366.0
A	75.3	3002.1	72.2	2717
T	28.7	2158.9	51.9	2354.1
G	79.2	2581.9	52.4	2134
C	57	1842.6	44.6	1493.8

### Gene ontology and amino acid distribution

**Fig. 1:**
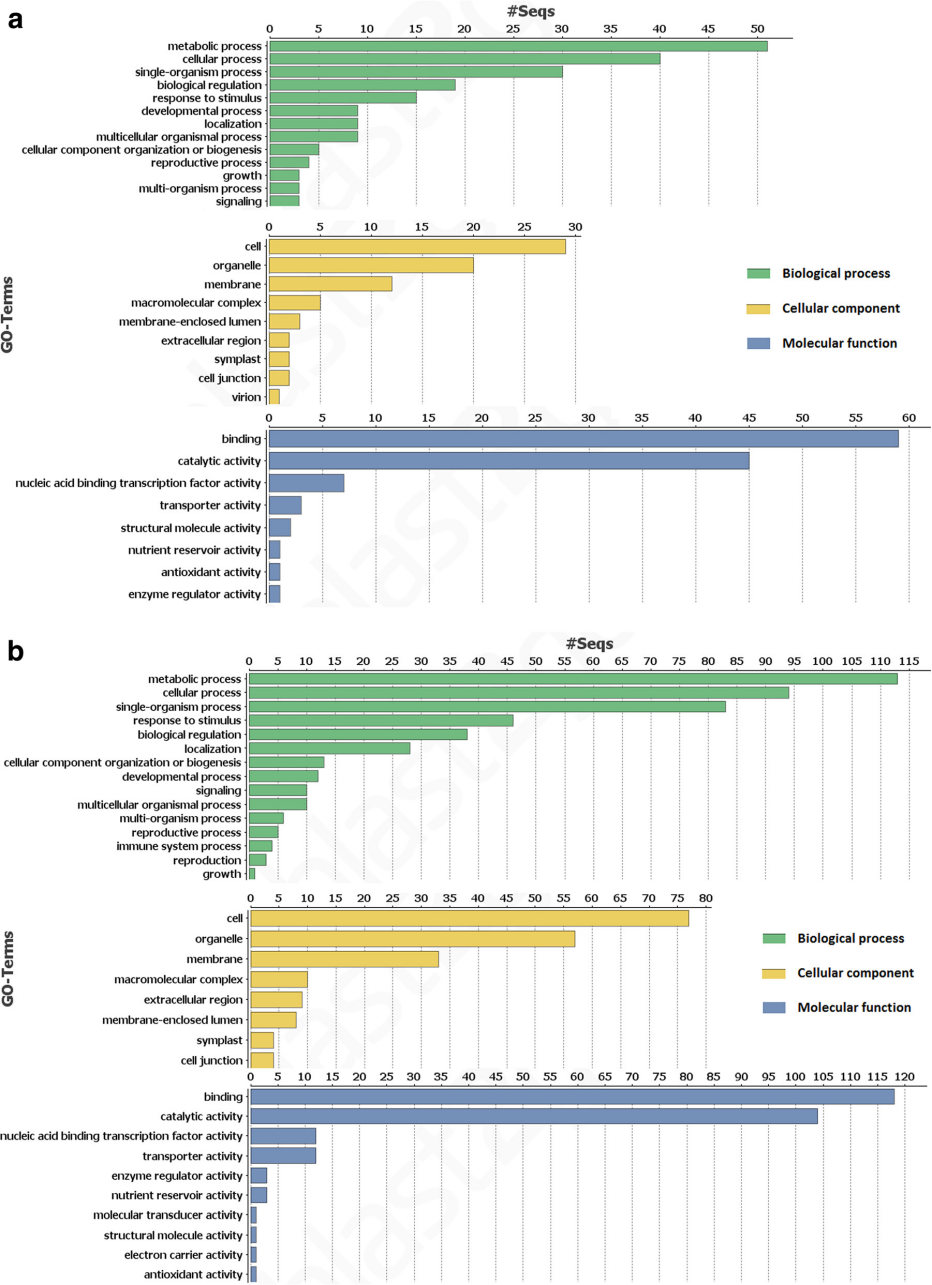
GO distribution by Level 2: Distribution of functional annotations among SSR containing genes in *Picea abies* and *Pinus taeda*. Results are summarized for three main GO categories: a) biological process, b) cellular component and c) molecular function. **a** *Picea abies*. **b** *Pinus taeda*

**Fig. 2:**
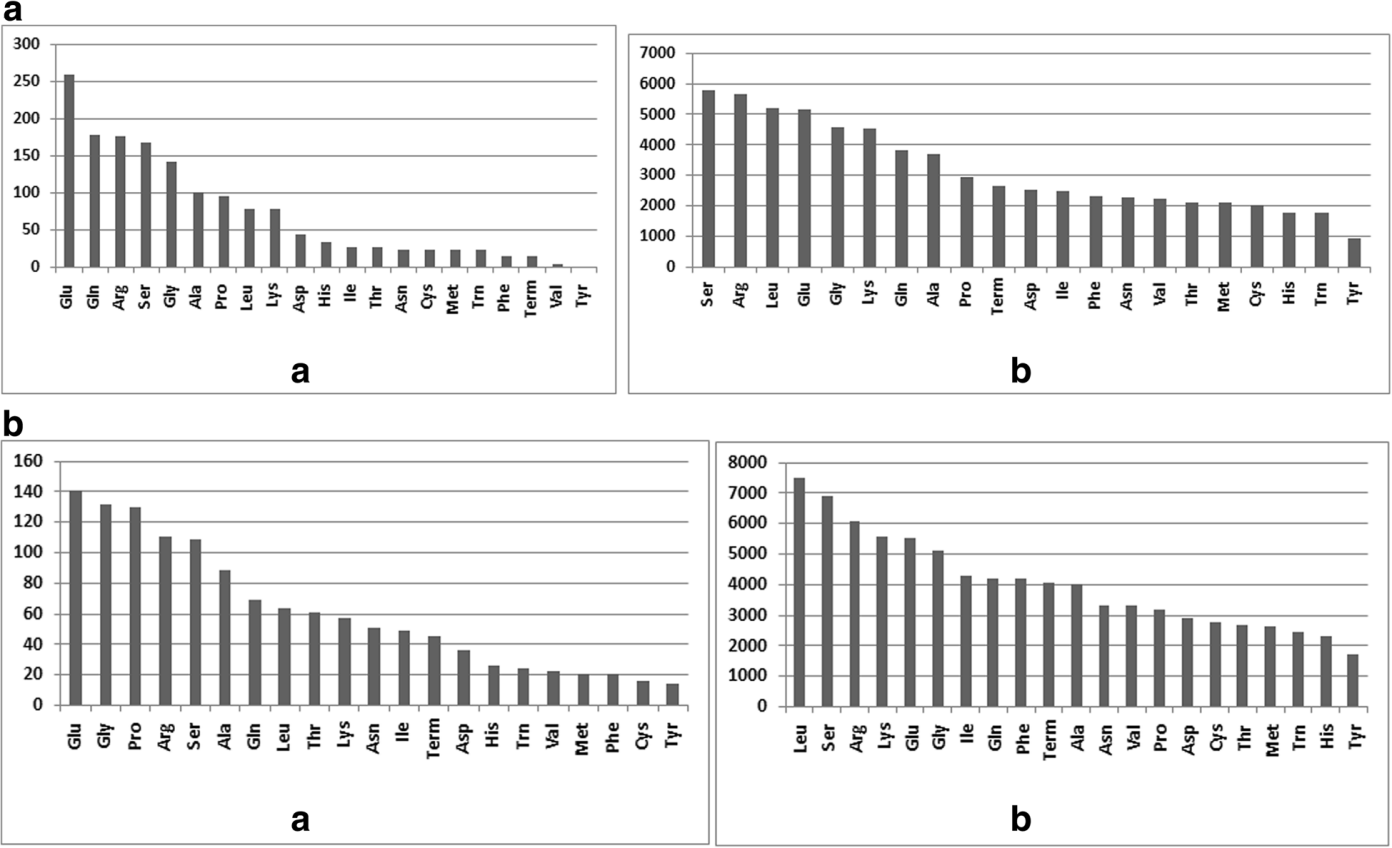
Amino acid occurrences in SSR loci in *Picea abies* and *Pinus taeda*: a) Class I SSRs b) Class II SSRs. **a** *Picea abies*. **b** *Pinus taeda*

The GO distribution of functional annotations in both species shows that the highest number of genes containing class I SSRs represent metabolic process, cell and binding for three main GO categories respectively (Fig. 1). Glutamic acid (Glu) is the most frequently occurring amino acid among the class I SSR loci in both species. With reference to class II SSRs, Serine (Ser) is the most commonly occurring amino in *Picea abies*, while Leucine (Leu) was most common in *Pinus taeda* (Fig. 2).

## Discussion

We have considered the high confidence full length coding regions of genes for the SSR analysis for the first time in gymnosperm species, while all the earlier studies involving gymnosperms have been carried out on ESTs. In addition, previously applied methodology also differs from ours (reviewed by [14]), e.g. some studies have considered 5′ UTR, ORF and 3′ UTR separately [14], while some have considered only 5′ESTs and 3′ESTs [15]. In the current study we have also analysed the class I and class II separately.

### Overall abundance of SSRs in Picea abies

Counts per Mbp SSR motifs were higher in *Picea abies* (Table 1), which is in partial agreement with earlier investigations [14, 15, 19] considering that in the current study the difference in counts per Mbp SSR motifs between the two species was significant only for class II SSRs. The motif length detected in the current study (class I SSRs) was lower as compared to the earlier studies in both genera [14, 18], but it is noteworthy that the standard error reported in the current study is also very low. In *Picea abies*, the overall abundance of SSR loci in class I is primarily the result of a higher frequency of trimers, which is three times higher compared to *Pinus taeda* (count per Mbp of hexamers in both species is similar – Table 2), whereas the higher frequency of SSRs in class II in *Picea abies* is largely as a result of additive effect of trimers and hexamers. This is again not in favour of an earlier study where the count per Mbp of trimers in both species was similar whereas the count per Mbp of hexamers was higher in *Pinus taeda* [19].

### Frequency of dimer motifs

Dimers were not detected in the class I SSR type in *Picea abies* and although were detected in the class II SSRs, they were not the most abundant types as found previously [14, 15, 18]. In a broader view, dimers are more frequent in lower plant species (algae and mosses), while trimer motifs are more frequent for the majority of higher plant groups (flowering plants) [18]. With reference to *Picea abies*, higher abundance of dimers was detected in EST-SSRs, but the majority of the studies were conducted on *Picea* spp. [15, 19, 24]. The only study conducted on *Picea abies* detected trimers (trimers > pentamers > hexmers) as the most abundant repeat [16]. Therefore, either the trimer frequency is species specific or the analysis is dependent on the data source involved and the parameters used for the detection of SSR repeats. In *Pinus taeda* on the other hand, trimers were most frequently detected in *Pinus* spp. [25], while the majority of the studies involving *Pinus taeda* [15, 18, 19], except one [17], showed dimers as the most abundant repeats. In our study, dimers represented the most abundant motifs after hexamers and trimers in class I SSRs, while it was the least detected category of SSR repeats in class II (Table 2). Overall, trimers were the most abundant motifs together with dimers in most of the studies in both species [15, 17, 19, 24]. Previously, it was reported that although a higher abundance of dimers was detected in EST-SSRs, the proportion of dimers to trimers decreased significantly in the ORF fraction in the majority of the genera including both angiosperm and gymnosperm species [14]. The sequence data is being updated continuously with recent advancements and as explained earlier, the use of a different sequence dataset for the SSR analysis is the most likely reason for not finding dimers as the most abundant motifs in both species.

### Trimers and hexamers are the most abundant motif types

Genome wide studies conducted to estimate the SSR distribution in eukaryotes reveal abundance of trimers and hexamers in the coding regions in lower single cellular organisms e.g. yeast [31] as well as higher organisms e.g. model plant systems like *Arabidopsis* [32, 33] and also in more complex organisms like human beings [34]. Trimers and hexamers are predominant as they are favoured by the selective pressures compared to the other repeats (e.g. dimers, tetramers and pentamers) considering that they do not alter the coding frame due to frameshift unless the length of the indel is divisible by three, e.g. in case of dimers an addition of three repeat motifs (e.g. ATATAT) will not modify the reading frame [35].

Although trimers were the most frequent motifs detected in the class I category, hexamers ranked as the next most abundant motifs in this class in *Picea abies*, while in *Pinus taeda* trimers and hexamers were equally abundant (Table 2). It is noteworthy that in *Picea abies* the proportion of trimers to hexamers in the same class is 3.1. The higher and lower proportion of trimers to hexamers in *Picea* and *Pinus taeda*, respectively, is similar to what has been reported by Berube et al. [15], but contrasts with the recent comparative study where the proportion of trimers to hexamers was lower in *Picea* spp. (1.5) and slightly higher in *Pinus* (1.3) [14]. Hexamers were the most abundant among the class II SSR types in both species and their count per Mbp was very high as compared to the other motif types. Predominance of trimers in *Picea abies* [16] and *Pinus taeda* [17] was reported earlier only in two studies, likewise Yan et al. [25] demonstrated higher frequency of trimers it in *Pinus* spp. Abundance of hexamers in gymnosperms is in accordance with earlier results in *Picea* [15, 16], *Pinus* [15], and Cryptomeria [36], as well as in comparative studies, which report hexamers to be more common among EST-SSRs in gymnosperms than angiosperms [14, 18]. The estimation of hexamer repeats was however under-estimated in earlier studies [14, 15], as a consequence of analysing only class I SSRs, whereas the current analysis reveals that there is very high abundance of hexamer repeats if class II SSRs are also taken into consideration (1100 and 971 per Mbp in spruce and pine, respectively).

Similar to previous investigations, AAT/ATT was one among the most frequent class I trimers in *Pinus taeda* [19] (Table 3). AAG/CTT was also one among the most abundant trimers, which was reported as the most frequent trimer in other studies in *Pinus* [17, 25] closely followed by ACG/CGT and AGG/CCT [17]. AGG/CCT and ACG/CGT were the most frequent trimer motifs within the class I category in *Picea abies*, which is similar to our previous results in the ORF fractions of *Picea* [14]. ACG/CGT was also the most abundant trimer detected by Berube et al. [15] in *Picea* and *Pinus taeda*. AAG/CTT motif was among the most abundant trimer repeats in class II SSRs of both species and class I SSRs of *Pinus taeda*, which was reported to be the second most frequent in *Pinus* and third most frequent in *Picea* within the class I trimers [14]. It is noteworthy that AGG/CCT and ACG/CGT are the trimer repeats detected in class I and class II as the most and equally abundant motifs among the others in both species.

### Frequency of AT-rich and GC-rich motifs

Abundance of AT-rich motifs was detected in class II SSRs in both species, which is in agreement with earlier studies in conifers [14, 15] (Table 5). Equal frequency of AT-rich and GC-rich motifs were found in class I SSRs of *Pinus taeda* while class I SSRs in *Picea abies* showed higher abundance of GC-rich motifs in contrast to earlier reports [14, 15]. This could be attributed to the difference in the data source considered, as the method used for detection of SSRs was similar as our previous study [14]. AT-rich segments in the coding region regulate DNA replication [37], while GC-rich elements in the coding region play important role in gene regulation [38].

### GO annotation

Among genes containing class I SSRs in both species, GO distributions show that the highest numbers of genes belong to the metabolic process, cell and binding, respectively for three main GO categories (Fig. 1). Similar results were reported in *Physcomitrella patens* and *Arabidopsis thaliana* [18]. However, the GO term with the highest number of genes containing SSR loci in Cryptomeria [36] was cellular process instead of metabolic process as is the case in *Pinus taeda* and *Picea abies*. Therefore, we suggest that the GO distribution may be species specific rather than generalised for gymnosperms as such.

Among class I SSR loci, glutamine (Glu) is the most represented amino acid in both conifer species studied (Fig. 2). In contrast, serine (Ser) was found to be the most frequent in *Gnetum* while arginine (Arg) was the most frequent in *Pinus taeda* [18]. In class II, Ser is the most frequent amino acid followed by Arg and leucine (Leu) in *Picea abies*, while Leu ranks first, followed by Ser and Arg in *Pinus taeda*. It is worth noticing that tyrosine (Tyr) ranks last in all cases. In this context, Glu and Ser repeats are amongst the few single amino acid repeats which are incorporated into many proteins to a considerable extent [39] and polyserine repeats are the most abundant in *Arabidopsis* [40].

## Conclusions

While several previous studies were based on EST datasets, for the first time in conifers, we report SSR loci in high confidence coding regions, which provides information on functional molecular markers that can be applied to genetic studies in *Pinus* and *Picea* species having prime economical and ecological importance. This analysis reveals an overall higher frequency of microsatellite repeats per Mbp in *Picea abies* as compared to *Pinus taeda*. It also supports abundance of hexamers in conifers. Although AT-rich and GC-rich repeats were equally abundant in *Pinus taeda*, GC-rich were found to be common in *Picea abies* in the class I SSR category.

### Availability of supporting data

All the supporting data are included as additional files.

## Additional file

Additional file 1:
**Putative primer pairs for the class I and class II SSRs in the coding regions of Norway spruce **(***Picea abies*** **) and Loblolly pine (** ***Pinus taeda*** **).** (XLSX 4157 kb)

## Abbreviations

SSR: Simple sequence repeats
CDS: Coding sequence
GO: Gene ontology
EST: Expressed sequence tags
UTR: Untranslated region
ORF: Open reading frame
